# Respiratory morbidity through the first decade of life in a national cohort of children born extremely preterm

**DOI:** 10.1186/s12887-018-1045-7

**Published:** 2018-03-07

**Authors:** Kaia Skromme, Maria Vollsæter, Knut Øymar, Trond Markestad, Thomas Halvorsen

**Affiliations:** 1 Department of Pediatrics, Haukeland University Hospital, N-5021 Bergen, Norway; 2 Department of Clinical Science, Faculty of Medicine and Dentistry, University of Bergen, Bergen, Norway; 3 Department of Pediatrics, Stavanger University Hospital, Stavanger, Norway

**Keywords:** Extremely preterm, Extremely low birth weight, Asthma, Hospitalisation, Respiratory health

## Abstract

**Background:**

Advances in perinatal care have markedly increased the prospects of survival for infants born extremely preterm (EP). The aim of this study was to investigate hospitalisation rates and respiratory morbidity from five to 11 years of age in a prospective national cohort of EP children born in the surfactant era.

**Methods:**

This was a national prospective cohort study of all children born in Norway during 1999 and 2000 with gestational age (GA) < 28 weeks or birth weight < 1000 grams, and of individually matched term-born controls recruited for a regional subsample. Data on hospital admissions, respiratory symptoms, and use of asthma medication was obtained by parental questionnaires at 11 years of age.

**Results:**

Questionnaires were returned for 232/372 (62%) EP-born and 57/61 (93%) regional term-born controls. Throughout the study period, 67 (29%) EP-born and seven (13%) term-born controls were admitted to hospital (odds ratio (OR) 2.90, 95% confidence interval (CI): 1.25, 6.72). Admissions were mainly due to surgical procedures, with only 12% due to respiratory causes, and were not influenced by neonatal bronchopulmonary dysplasia (BPD) or low GA(≤ 25 weeks). Respiratory symptoms, asthma and use of asthma medication tended to be more common for EP-born, significantly so for medication use and wheeze on exercise. Neonatal BPD was a risk factor for medication use, but not for current wheeze. In multivariate regression models, home oxygen after discharge (OR 4.84, 95% CI: 1.38, 17.06) and parental asthma (OR 4.38, 95% CI: 1.69, 11.38) predicted current asthma, but neither BPD nor low GA were associated with respiratory symptoms at 11 years of age.

**Conclusions:**

Hospitalisation rates five to 11 years after EP birth were low, but twice those of term-born controls, and unrelated to neonatal BPD and low GA. Respiratory causes were rare. Respiratory complaints were more common in children born EP, but the burden of symptoms had declined since early childhood.

## Background

Since the early 1990s increasing numbers of infants born extremely preterm (EP) in high-income countries have survived [1, 2]. Birth at this stage of pregnancy interrupts important developmental processes, and requires gas exchange to take place in foetal lungs, often leading to the syndrome of bronchopulmonary dysplasia (BPD) [3]. The life-long health consequences of EP birth and BPD are unknown, but there are concerns of severe future morbidities, such as chronic obstructive pulmonary disease [4], metabolic syndrome [5], cardiovascular diseases and even early death [6, 7]. Continued health surveillance is therefore important in this group, particularly for those born at less than 26 weeks gestational age (GA), as their high survival rates are fairly recent history.

Health problems may be reflected in utilisation of health care services. Children born EP more often experience repeated hospital admissions during early childhood than children born at term [8]. Most published data on later outcome pertain to groups born in the pre-surfactant era, and there is a need for population based knowledge on health issues among EP-born survivors exposed to the advanced treatment facilities of the late 1990s and 2000s. Such data are of interest to a growing part of health care professionals, administrators, politicians, the EP-born individuals themselves and their families.

We have previously published data on morbidities and hospital admissions during the first five years of life in a national cohort of EP-born children [9, 10]. The aims of the present study were to investigate frequencies and causes of hospital admissions, general health issues and early predictors of health at five to 11 years of age in that same cohort, with a particular focus on respiratory outcomes.

## Methods

### Participants

All subjects born EP, here defined as GA 22^0^ to 27^6^ weeks or birth weight 500 to 999 grams, in Norway during 1999 and 2000 were included at birth and followed prospectively during their stay at the neonatal intensive care unit (NICU) [2] and at two [11], five [9, 10] and 11 years of age [12]. Of 638 eligible infants, 174 were stillborn or not resuscitated, 464 were admitted to a NICU and 372 (80%) were alive at 11 years of age.

A control group was recruited at 11 years of age for a regional subsample of participants born EP within Western Norway Regional Health Authority (*n* = 61) by inviting the next-born child of the same gender with GA > 37 weeks and birth weight (BW) > 3000 grams, identified from birth protocols at the maternity ward [12]. If that individual declined, the next-born eligible child was invited until a match was obtained.

The study was based on written parental consent and was approved by the Regional committee on Medical Research Ethics and the Norwegian Data Inspectorate.

### Data collection

**Fig. 1:**
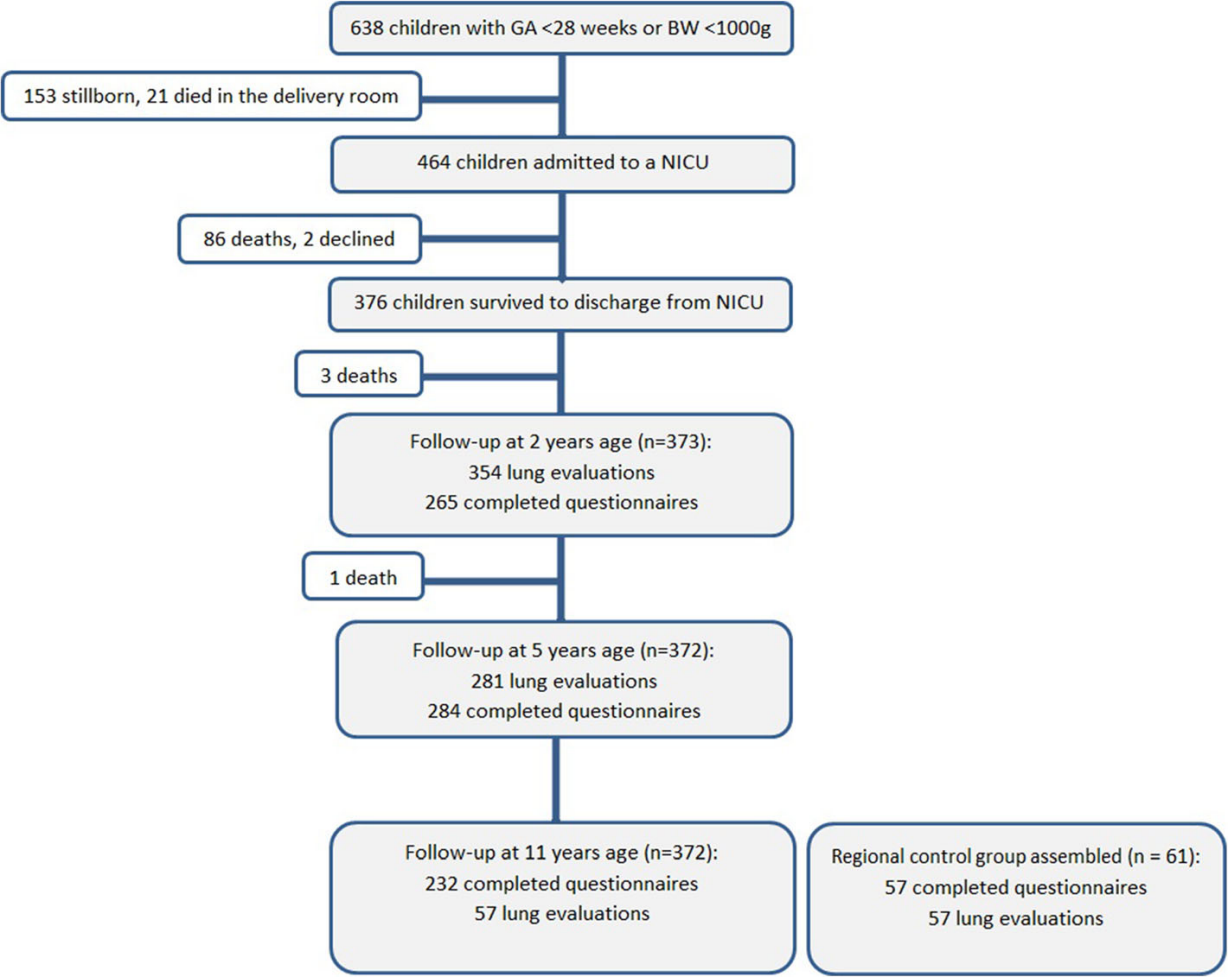
Description of the Nationwide Cohort of Children Born in Norway During 1999 and 2000 at a Gestational Age <28 Weeks or with a Birth Weight <1000 Grams. *Abbreviations:* GA – Gestational Age, BW – Birth Weight, NICU – Neonatal Intensive Care Unit

For the children born EP, all obstetric and paediatric departments in Norway participated in collecting data on the neonatal course and follow-up at two and five years of age, as illustrated in Fig. 1, which explains the recruitment and follow-up process of the overall study. Data on maternal health, pregnancy, delivery and NICU stay were extracted from compulsory notifications to the Medical Birth Registry of Norway. All the data were registered prospectively using forms developed for this study [2]. The children were examined by experienced paediatricians at two and five years of age, and the parents completed questionnaires on socio-demographic factors, health, development, and hospital admissions at two, five and 11 years of age. The International Study of Asthma and Allergies in Childhood (ISAAC) questionnaire was used at both five and 11 years of age to collect data on respiratory health, as well as to compare the burden of respiratory symptoms over this timespan.

Respiratory health and hospital admissions up to five years of age have been published previously [10] and are used as background data in the current article, which reports data from five to 11 years of age. The data were collected by postal questionnaire completed by the parents when the child was 11 years old. Within the region of Western Norway the children were also examined clinically at 11 years of age, together with their matched term-controls [12].

### Definitions

GA at birth was based on the national antenatal care program that includes one ultrasound examination at 17–18 weeks gestation, except for a few participants (5%) for whom GA relied on the last menstrual period because an ultrasound was not performed. Small for gestational age was defined as a BW less than the fifth percentile for GA and gender according to Norwegian growth curves [13]. Premature rupture of membranes was defined as rupture more than six days prior to labour. Prenatal steroids were recorded if given at least 24 hours before delivery or at least as two doses. BPD was defined as need of assisted ventilation or oxygen supplementation at 36 completed post-menstrual weeks [3].

At five years of age, major neurosensory disability was defined as cerebral palsy, blindness (legally classified as blind) or complete deafness. For the mothers, a minimum of three years of college education or a university degree was classified as higher education. Cerebral ultrasound findings were dichotomised to minor and major pathology, i.e. respectively periventricular haemorrhage grade 1 to 2, or a maximum of two small cysts, versus periventricular haemorrhage grade 3 to 4 or multicystic periventricular leukomalacia.

Respiratory illness as a cause for admission to hospital included airway infections and all kinds of breathing problems. Current wheeze at 11 years of age was defined by parental report during the past 12 months, while current asthma was defined as either a doctor’s diagnosis of asthma combined with either respiratory symptoms or use of asthma medication in the previous 12 months, or asthma medication and symptoms in the past 12 months even if no recall of prior doctor’s diagnosis. According to Lai et al. [14], severe asthma was defined as four or more episodes of wheezing, or sleep disturbances (awakened more than once a week) or problems of speaking due to wheezing during the past 12 months. Asthma medication included inhaled corticosteroids, short or long acting β_2_-agonists and oral leukotriene modifiers.

### Statistical methods

Data were presented as means with standard deviations or as medians with interquartile ranges. Group comparisons were performed with the Student’s t-test, χ^2^ test, Fisher’s exact test or Mann-Whitney U-test, as appropriate. Changes in respiratory health measures were analysed using related samples McNemar’s test, and point estimates with 95% confidence intervals (95% CI) were also reported to account for the children with missing responses between questionnaires.

Risk factors for admissions to hospital and for having current asthma were assessed with binary logistic regression, and results expressed as odds ratios (OR) with 95% CI. Neonatal and socio-demographic variables entered in the analyses are listed in the first column of Table 4. Multivariate risk models were constructed by entering all variables with a *p*-value < 0.10 in univariate regression analyses. *P*-values ≤0.05 were considered significant. As multiple hypotheses were tested in regression models utilising a dataset with limited numbers of participants, Bonferroni corrections were performed and reported in the Results section. All analyses were conducted with SPSS software version 22.0 for Windows.

## Results

Questionnaires were returned for 232 (62%) of the 372 surviving children at 11 years of age. Corresponding figures at two and five years of age were 265 (71%) and 284 (76%), respectively. The ISAAC questionnaire was completed for 192 of the children at both five and 11 years of age. Table 1 accounts for differences between participants and non-participants at 11 years of age. The median GA, proportion of mothers with higher education, and proportion of infants who received surfactant and had BPD were higher among the participants, while proportions of mothers who smoked during pregnancy or had chorioamnionitis, and of boys, retinopathy of prematurity or major neurosensory disability at five years of age were lower.

**Table 1:** Early Characteristics of Extremely Preterm Children Born in Norway During 1999 and 2000 at a Gestational Age < 28 Weeks or with a Birth Weight < 1000 Grams, According to Whether Response Was Given to the Parental Questionnaire at 11 Years of Age

	Parental questionnaire at 11 years of age^a^		
	Responders *n* = 232	Non-responders *n* = 140	*p*-value^b^
Gestational age, median (interquartile range)	27 (26–28)	26 (25–27)	**0.014**
Birth weight, median (interquartile range)	880 (766–994)	847 (715–979)	0.376
Illness severity score^c^, median (interquartile range)	1 (0–2)	1 (0.5–1.5)	0.063
Length of initial stay, median (interquartile range)	92 (70–114)	89 (67–111)	0.483
Male sex	115/232 (50%)	85/140 (61%)	**0.037**
Caesarean section	159/232 (69%)	84/140 (60%)	0.094
Mother higher education	109/227 (48%)	40/120 (33%)	**0.009**
Premature rupture of membranes^d^	26/217 (12%)	13/125 (10%)	0.658
Multiple births	56/232 (24%)	27/140 (19%)	0.276
Bronchopulmonary dysplasia^e^	113/232 (49%)	52/140 (37%)	**0.030**
Patent ductus arteriosus	93/232 (40%)	50/140 (36%)	0.401
Small for gestational age^f^	46/232 (20%)	23/140 (16%)	0.414
Sepsis	52/232 (22%)	36/140 (26%)	0.468
Cerebral ultrasound findings^g^			
No pathology	152/232 (66%)	94/140 (67%)	0.814
Minor pathology	61/232 (26%)	33/140 (24%)	
Major pathology	19/232 (8%)	13/140 (9%)	
Prenatal steroids	164/232 (71%)	93/140 (66%)	0.389
Postnatal steroids	82/232 (35%)	47/140 (34%)	0.728
Mother’s age, mean (standard deviation)	30 (5)	29 (6)	0.280
Chorioamnionitis	32/232 (14%)	35/140 (25%)	**0.006**
Preeclampsia	56/232 (24%)	34/140 (24%)	0.974
Retinopathy of prematurity	60/232 (26%)	52/138 (38%)	**0.017**
Smoking in pregnancy	46/191 (24%)	47/124 (38%)	**0.009**
Cerebral palsy, blind or deaf at five years of age	13/232 (6%)	20/140 (14%)	**0.004**
Home oxygen treatment	22/231 (10%)	9/124 (7%)	0.471
Surfactant	192/232 (83%)	104/140 (74%)	**0.050**
Lung disease at two years of age	62/223 (28%)	45/131 (34%)	0.195

^a^Figures are given as n (%), unless otherwise specified. Percentages were calculated from the actual response rates that varied slightly between the items

^b^Mann Whitney’s U test, Student’s T-test, or χ^2^ test, as appropriate. Boldface denotes significant group differences

^c^A score based on lowest and highest fractional oxygen (FIO_2_) requirements and the base deficit during the first 12 hours of life

^d^Defined as rupture of membranes more than  six days before delivery

^e^Defined as assisted ventilation or oxygen supplementation at 36 weeks postmenstrual age

^f^Defined as less than the fifth percentile for GA and gender according to Norwegian growth curves

^g^Minor pathology defined as periventricular haemorrhage grade 1 to 2, or a maximum of two small cysts, and major pathology defined as periventricular haemorrhage grade 3 to 4 or multi-cystic periventricular leukomalacia

On average, 1.6 term-born subjects had to be approached to recruit one consenting match for each of the 61 eligible subjects born EP within Western Norway Regional Health Authority. Questionnaires were returned for 57 (93%) of the control children.

### Hospital admissions

**Fig. 2:**
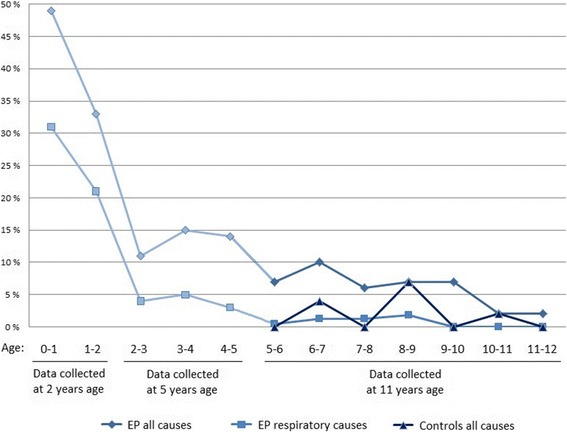
Hospital Admissions from Birth to Mid-Childhood in Children Born in Norway During 1999 and 2000 at a Gestational Age < 28 Weeks or with a Birth Weight < 1000 Grams, Split by All Causes and Respiratory Causes. Also Depicted are Admissions for All Causes from Age Five to 11 Years of Age for a Regional Control Group Assembled at 11 Years of Age. *Abbreviations*: EP – Extremely Preterm

The overall admission rate from five to 11 years of age was significantly higher for children born EP than the term-born controls (OR 2.90, 95% CI: 1.25, 6.72). The proportions of readmitted children from birth to 11 years of age are presented in Fig. 2. There were no significant differences in admission rates between those with and without BPD or between GA categories (Table 2). The 13 children with major neurosensory disability (6% of participants) accounted for 32/138 (23%) of all admissions, and 21% of the children with more than one admission. The admission rate was significantly lower during 5–11 years of age than during the 0–5 year period (29%, 95% CI: 23–35% versus 75%, 95% CI: 70–80%, *P* < 0.001).

**Table 2:** Rates and Numbers of Admissions to Hospital at Five to 11 Years of Age in Children Born in Norway During 1999 and 2000 at a Gestational Age < 28 Weeks or with a Birth Weight < 1000 Grams and a Regional Control Group Assembled at 11 Years of Age

	All participants			Neonatal bronchopulmonary dysplasia^b^			Gestational age in weeks^c^				
	Cases *n* = 232	Controls *n* = 57	*p-*values ^d^	Yes *n* = 165	No *n* = 207	*p-*values ^d^	≤25 *n* = 99	26–27 *n* = 188	≥28 *n* = 85	*p-*values^d^ ≤ 25 vs. 26–27	*p-*values^d^ 26–27 vs. ≥28
Admitted 5–11 years of age^a^											
Admitted	67 (29%)	7 (13%)	**0.010**	34 (31%)	33 (28%)	0.597	18 (36%)	31 (26%)	18 (30%)	0.193	0.576
Admitted for respiratory cause	8 (4%)	1 (2%)	1.000	6 (6%)	2 (2%)	0.158	3 (6%)	3 (3%)	2 (3%)	0.362	1.000
Admissions^e^	138	7	**0.006**	77	61	0.451	36	64	38	0.192	0.482
Admissions for respiratory cause	16 (12%)	1 (14%)	0.507	14 (41%)	2 (6%)	0.117	6	8	2	0.273	0.773
Number of admissions per child											
Once	38 (57%)	7 (100%)	0.083	17 (50%)	21 (64%)	0.355	11 (61%)	19 (61%)	8 (44%)	0.659	0.463
Twice	14 (21%)	0		7 (21%)	7 (21%)		2 (11%)	6 (19%)	6 (33%)		
Three times or more	15 (22%)	0		10 (29%)	5 (15%)		5 (28%)	6 (19%)	4 (22%)		

^a^Figures are numbers of admitted children (% of group) and admissions

^b^Defined as assisted ventilation or oxygen supplementation at 36 weeks postmenstrual age

^c^For analysis regarding gestational age, the fraction born at 26–27 weeks were selected as the reference category (those with gestational age ≥ 28 had birth weights < 1000 grams)

^d^Independent samples Mann-Whitney’s U Test or χ^2^ test, as appropriate. Boldface denotes significant group differences

^e^Information on admissions was given for 229 subjects

Surgery was the most common reason for admissions (Table 3), and adeno-tonsillectomies and insertion of ear ventilation tubes were significantly more common among EP-born children than controls (*p* = 0.033). Of the 67 children admitted during the study period, 45 (67%) had also been admitted before five years of age. By 11 years of age, parents of 213/372 children (57% of the total cohort) had reported a hospital admission in at least one of the three questionnaires (at two, five or 11 years of age), and 121/372 children an admission for respiratory causes (33% of the total cohort).

**Table 3:** Causes for Admission to Hospital at Five to 11 Years of Age for Extremely Preterm Children Born in Norway During 1999 and 2000 at a Gestational Age < 28 Weeks or with a Birth Weight < 1000 Grams and a Regional Control Group Assembled at 11 Years of Age

Admission causes^a^	Cases *n* = 138	Controls *n* = 7
Respiratory causes	16 (12%)	1 (14%)
Surgery	69 (50%)	6 (86%)
Hernia repairs	3	1
Adeno−tonsillectomy or ear ventilation tube insertion	31	1
Central nervous system	2	
Gastrointestinal	4	
Orthopedic procedures	13	3
Plastic surgery	4	1
Eye surgery	9	
Male genitalia	3	
Gastrointestinal	9 (7%)	0
Central nervous system	27 (20%)	0
Nutritional	3 (2%)	0
Other	2 (1%)	0
Unknown^b^	12 (9%)	0

^a^Figures are number of admissions (% of total)

^b^Parents that reported the number of admissions for their child, but failed to specify the causes

In multivariate regression models, a *higher* GA at birth and the presence of major neurosensory disability at five years of age were significantly associated with admission during the study period, while birth by caesarean section was a significant protective factor (Table 4). Adjusted for multiple hypotheses being tested, disability at five years of age and caesarean section were closest to reaching the significance limit (Bonferroni adjusted *p*-value 0.056 and 0.140, respectively). Removing children with major neurosensory disability at five years of age from the analysis did not alter the effect of the other variables. When added to the model, hospital admission during the third to fifth year of life was significantly associated with admission between five to 11 years of age (OR 3.48, 95% CI:1.60, 7.56), but did not alter the effect of other variables.

**Table 4:** Hospital Admissions at Five to 11 Years Age and Current Respiratory Symptoms and Current Asthma at 11 Years of Age According to Perinatal and Socio-Demographic Characteristics in Unadjusted and Adjusted Logistic Regression Models for 232 Extremely Preterm Children Born in Norway During 1999 and 2000 at a Gestational Age < 28 Weeks or with a Birth Weight < 1000 Grams

	Admitted to hospital 5–11 years of age		Respiratory symptoms past 12 months at 11 years of age		Current asthma at 11 years of age	
Characteristic	Unadjusted model OR (95% CI)	Adjusted model OR (95% CI)	Unadjusted model OR (95% CI)	Adjusted model OR (95% CI)	Unadjusted model OR (95% CI)	Adjusted model OR (95% CI)
Gestational age	1.00 (0.84, 1.19)	1.33 (1.04, 1.71)*	0.91 (0.73, 1.13)	1.00 (0.77, 1.29)	0.82 (0.65, 1.03)	0.88 (0.65, 1.19)
Birth weight/ 50 grams	1.04 (0.96, 1.14)		1.00 (0.90, 1.12)		0.98 (0.88, 1.09)	
Mother’s age / five years	0.76 (0.57, 1.01)	0.75 (0.55, 1.02)	0.57 (0.40, 0.83)**	0.57 (0.39, 0.84)**	0.77 (0.54, 1.10)	
Chorioamnionitis	1.81 (0.84, 3.92)		2.40 (1.01, 5.73)*	1.97 (0.72, 5.42)	2.51 (1.05, 5.99)*	1.76 (0.60, 5.13)
Preeklampsia	0.59 (0.29, 1.20)		1.20 (0.54, 2.66)		0.88 (0.38, 2.06)	
PROM^a^	0.91 (0.36, 2.30)		1.39 (0.48, 3.98)		0.45 (0.10, 1.99)	
Sepsis	0.76 (0.38, 1.54)		1.35 (0.61, 3.01)		1.66 (0.76, 3.66)	
Prenatal steroids	1.18 (0.63, 2.23)		0.98 (0.45, 2.11)		0.69 (0.33, 1.46)	
Caesarean section	0.50 (0.28, 0.92)*	0.32 (0.15, 0.71)**	0.82 (0.39, 1.72)		0.78 (0.37, 1.64)	
Multiple birth	1.86 (0.97, 3.54)	1.65 (0.78, 3.49)	0.85 (0.36, 1.97)		0.58 (0.23, 1.49)	
Male sex	1.43 (0.81, 2.54)		1.61 (0.79, 3.29)		1.02 (0.50, 2.08)	
Small for gestational age^b^	0.94 (0.46, 1.93)		0.75 (0.29, 1.92)		0.61 (0.22, 1.67)	
Surfactant	1.06 (0.50, 2.28)		1.09 (0.42, 2.82)		1.35 (0.49, 3.71)	
Postnatal steroids	1.66 (0.92, 2.99)	1.82 (0.80, 4.12)	0.86 (0.41, 1.81)		1.38 (0.67, 2.84)	
Patent ductus arteriosus	0.74 (0.41, 1.34)		1.02 (0.50, 2.09)		1.41 (0.69, 2.89)	
Retinopathy of prematurity	1.08 (0.57, 2.07)		1.47 (0.69, 3.15)		1.54 (0.72, 3.32)	
Illness severity score^c^	1.16 (1.01, 1.33)*	1.13 (0.96, 1.31)	1.11 (0.94, 1.30)		1.16 (0.99, 1.36)	1.16 (0.96, 1.41)
Length initial stay / seven days	1.01 (0.98, 1.06)		1.03 (0.98, 1.07)		1.04 (0.99, 1.09)	1.00 (0.94, 1.07)
Bronchopulmonary dysplasia^d^						
Without home oxygen	0.96 (0.52, 1.78)	0.78 (0.37, 1.65)	1.02 (0.48, 2.18)		1.44 (0.66, 3.16)	0.92 (0.36, 2.39)
With home oxygen	2.37 (0.92, 6.10)	1.26 (0.39, 4.13)	1.65 (0.54, 5.04)		4.04 (1.38, 11.83)*	4.84 (1.38, 17.06)*
Cerebral ultrasound findings^e^	1.63 (0.88, 3.03)					
Minor pathology	1.28 (0.66, 2.45)	1.19 (0.57, 2.47)	1.30 (0.59, 2.88)		1.16 (0.51, 2.62)	
Major pathology	**2.54 (0.96, 6.71)**	0.76 (0.19, 2.99)	1.58 (0.48, 5.19)		1.58 (0.48, 5.19)	
Disability at five years	9.30 (2.47, 34.99)***	12.33 (2.48, 61.36)**	1.63 (0.43, 6.24)		2.60 (0.76, 8.94)	
Smoking in pregnancy	1.10 (0.53, 2.27)		1.61 (0.67, 3.87)		1.12 (0.44, 2.85)	
Smoking in home	0.66 (0.36, 1.21)		0.92 (0.44, 1.92)		0.91 (0.44, 1.92)	
Single parent	1.73 (0.78, 3.83)		1.31 (0.50, 3.45)		2.62 (1.09, 6.29)*	1.51 (0.63, 3.61)
Siblings < six years (yes/no)	0.71 (0.29, 1.72)		0.31 (0.070, 1.40)		0.61 (0.17, 2.18)	
Breast milk after discharge	1.25 (0.62, 2.53)		0.99 (0.42, 2.33)		0.65 (0.27, 1.55)	
Mother higher education	0.89 (0.50, 1.59)		1.45 (0.70, 3.02)		1.10 (0.52, 2.32)	
Parental history of asthma	0.73 (0.33, 1.65)		3.80 (1.71, 8.45)***	3.12 (1.33, 7.33)**	4.00 (1.79, 8.93)***	4.38 (1.69, 11.38)**

*Abbreviations: OR* odds ratio, *CI* confidence interval, *PROM* premature rupture of membranes

^a^Defined as rupture of membranes more than six days before delivery

^b^Defined as less than fifth percentile for GA and gender according to Norwegian growth curves

^c^A score based on lowest and highest fractional oxygen (FIO_2_) requirements and the base deficit during the first 12 hours of life

^d^Defined as assisted ventilation or oxygen supplementation at 36 weeks postmenstrual age

^e^Minor pathology defined as periventricular haemorrhage grade 1 to 2, or a maximum of two small cysts, and major pathology defined as periventricular haemorrhage grade 3 to 4 or multi-cystic periventricular leukomalacia

*p ≤ 0.05; **p ≤ 0.01; ***p ≤ 0.001. Boldface denotes significant group differences

### Respiratory health

A higher proportion of the EP-born than the term-born children had experienced wheezing and used asthma medications from five to 11 years of age, and a higher proportion of those born EP reported wheezing on exercise during the last 12 months at 11 years of age. There were no significant differences for the other ISAAC questions, but there was a general tendency towards more symptoms and treatments in the EP-born group (Table 5). However, there was a significant decline in the rates of wheezing, awakenings due to wheeze, dry cough at night, current asthma and current use of asthma medications from five to 11 years of age in the EP-born children (Table 6). The rates of parental asthma was similar for the EP- and term-born groups (17% versus 13%, *p* = 0.451), but a higher proportion of the children born EP lived in smoking households at 11 years of age (38% versus 23%, *p* = 0.038).

**Table 5:** Respiratory Health at Five to 11 Years of Age for Extremely Preterm Children Born in Norway During 1999 and 2000 at a Gestational Age < 28 Weeks or with a Birth Weight < 1000 Grams and a Regional Control Group Assembled at 11 Years of Age

	All participants			Neonatal Bronchopulmonary dysplasia^a^			Gestational age in weeks^b^				
	Cases *n* = 232	Controls *n* = 57	OR (95% CI)	Yes: *n* = 165	No *n* = 207	OR (95% CI)	≤25 *n* = 99	26–27 *n* = 188	≥28 *n* = 85	OR (95% CI) ≤25 vs. 26–27	OR (95% CI) 26–27 vs. ≥28
Birth to 11 years of age:											
Ever diagnosed with asthma	69 (30%)	5 (9%)	4.40 (1.69, 11.50)**	39 (35%)	30 (25%)	1.56 (0.89, 2.76)	19 (37%)	36 (30%)	14 (23%)	1.40 (0.70, 2.79)	0.72 (0.35, 1.47)
Ever used asthma medication	110 (47%)	7 (13%)	6.31 (2.74, 14.52)***	69 (61%)	41 (35%)	2.98 (1.75, 5.09)***	31 (61%)	61 (50%)	18 (30%)	1.53 (0.78, 2.97)	0.42 (0.22, 0.81)
From five to 11 years of age:											
Wheezing	60 (26%)	7 (13%)	2.51 (1.08, 5.83)*	36 (32%)	24 (20%)	1.83 (1.01, 3.33)*	14 (28%)	36 (30%)	10 (17%)	0.88 (0.43, 1.83)	0.47 (0.21, 1.02)
Asthma medication use	59 (27%)	3 (5%)	6.52 (1.96, 21.65)**	35 (34%)	24 (21%)	1.97 (1.08, 3.62)*	14 (30%)	34 (30%)	11 (19%)	1.03 (0.49, 2.17)	0.54 (0.25, 1.16)
Inhaled corticosteroids	45 (21%)	3 (6%)	4.51 (1.35, 15.11)*	30 (29%)	15 (13%)	2.68 (1.34, 5.33)**	9 (19%)	29 (26%)	7 (12%)	0.67 (0.29, 1.55)	0.41 (0.17, 0.99)*
Others	49 (22%)	3 (6%)	4.94 (1.48, 16.50)**	30 (29%)	19 (16%)	2.09 (1.09, 4.00)*	13 (28%)	28 (24%)	8 (14%)	1.25 (0.58, 2.70)	0.51 (0.22, 1.20)
LRTI treated with antibiotics	34 (15%)	2 (4%)	4.75 (1.11, 20.38)*	18 (16%)	16 (14%)	1.14 (0.55, 2.33)	9 (18%)	18 (15%)	8 (13%)	1.23 (0.51, 2.95)	0.88 (0.36, 2.16)
Last 12 months at 11 years of age											
Wheezing	37 (16%)	5 (9%)	1.97 (0.74, 5.27)	19 (17%)	18 (15%)	1.13 (0.56, 2.29)	10 (20%)	20 (17%)	7 (12%)	1.23 (0.53, 2.86)	0.67 (0.27, 1.68)
Number of attacks											
None	199 (86%)	52 (91%)	*P* = 0.511	97 (86%)	102 (86%)	*P* = 0.929	42 (82%)	104 (86%)	53 (88%)	*P* = 0.675	*P* = 0.776
1–3	18 (8%)	3 (5%)		10 (9%)	8 (7%)		4 (8%)	10 (8%)	4 (7%)		
4–12	7 (3%)	0 (0%)		3 (3%)	4 (3%)		3 (6%)	2 (2%)	2 (3%)		
> 12	5 (2%)	2 (4%)		2(2%)	3 (3%)		1 (2%)	3 (3%)	1 (2%)		
All the time	3 (1%)	0 (0%)		1 (1%)	2 (2%)		1 (2%)	2 (2%)	0 (0%)		
Wheeze on exercise	41 (18%)	3 (5%)	3.86 (1.15, 12.97)*	23 (20%)	18 (15%)	1.43 (0.73, 2.83)	11 (22%)	21 (17%)	9 (15%)	1.31 (0.58, 2.96)	0.84 (0.36, 1.97)
Problem speaking due to wheezing	2 (1%)	0 (0%)	NC	0 (0%)	2 (2%)	NC	0 (0%)	1 (1%)	1 (2%)	NC	2.03 (0.13, 33.11)
Dry cough at night	37 (16%)	4 (7%)	2.48 (0.85, 7.27)	18 (16%)	19 (16%)	1.01 (0.50, 2.04)	13 (26%)	17 (14%)	7 (12%)	2.15 (0.95, 4.85)	0.81 (0.32, 2.07)
Ever awakened due to wheezing	16 (7%)	1 (2%)	4.15 (0.54, 31.95)	8 (7%)	8 (7%)	1.06 (0.38, 2.92)	5 (10%)	8 (7%)	3 (5%)	1.54 (0.48, 4.94)	0.74 (0.19, 2.91)
LRTI treated with antibiotics	5 (2%)	1 (2%)	1.24 (0.14, 10.87)	1 (1%)	4 (3%)	0.26 (0.023, 2.33)	1 (2%)	3 (3%)	1 (2%)	0.80 (0.081, 7.84)	0.66 (0.067, 6.49)
Currently at 11 years of age											
Current asthma (criteria-based)^c^	36 (16%)	4 (7%)	2.43 (0.83, 7.14)	22 (20%)	14 (12%)	1.81 (0.88, 3.75)	12 (24%)	17 (14%)	7 (12%)	1.88 (0.82, 4.30)	0.81 (0.32, 2.07)
Severe asthma (criteria-based)^d^	18 (8%)	2 (4%)	2.31 (0.52, 10.27)	7 (6%)	11 (9%)	0.65 (0.24, 1.74)	6 (12%)	8 (7%)	4 (7%)	1.88 (0.62, 5.73)	1.01 (0.29, 3.49)
Asthma medication use	34 (15%)	3 (5%)	3.09 (0.91, 10.45)	22 (20%)	12 (10%)	2.16 (1.01, 4.60)*	10 (20%)	18 (15%)	6 (10%)	1.40 (0.59, 3.28)	0.64 (0.24, 1.70)
Inhaled corticosteroids	21 (9%)	3 (5%)	1.81 (0.52, 6.29)	15 (13%)	6 (5%)	2.89 (1.08, 7.73)*	5 (10%)	13 (11%)	3 (5%)	0.92 (0.31, 2.74)	0.44 (0.12, 1.63)
Bronchodilators	25 (11%)	3 (5%)	2.21 (0.64, 7.58)	16 (14%)	8 (5%)	2.04 (0.86, 4.83)	9 (18%)	11 (9%)	5 (9%)	2.18 (0.84, 5.63)	0.92 (0.30, 2.77)
Singulair	7 (3%)	0 (0%)	NC	4 (4%)	3 (3%)	1.43 (0.31, 6.55)	2 (4%)	4 (3%)	1 (2%)	1.20 (0.21, 6.82)	0.50 (0.055, 4.58)

*Abbreviations: OR* odds ratio, *CI* confidence interval, *LRTI* Lower respiratory tract infection, *NC* non-calculable

^a^Defined as assisted ventilation or oxygen supplementation at 36 weeks postmenstrual age

^b^For analysis regarding gestational age, the fraction born at 26–27 weeks were selected as the reference category (those with gestational age ≥ 28 weeks had birth weights < 1000 grams)

^c^Defined by either (1) a doctor’s diagnosis of asthma combined with either respiratory symptoms or use of asthma medication in the previous 12 months, or (2) asthma medication and symptoms in the past 12 months even if no recall of prior doctor’s diagnosis

^d^Defined by four or more episodes of wheezing, or sleep disturbances (awakened more than once a week), or problems of speaking due to wheezing reported during the past 12 months (14)

*p ≤ 0.05; **p ≤ 0.01; ***p ≤ 0.001. Boldface denotes significant group differences

**Table 6:** Respiratory Health the past 12 Months for Extremely Preterm Children Born in Norway During 1999 and 2000 at a Gestational Age < 28 Weeks or with a Birth Weight < 1000 Grams Assessed at Five and 11 Years of Age by the International Study of Asthma and Allergy in Childhood Questionnaire

	At 5 years of age *n* = 284	At 11 years of age *n* = 232	
	Rate (95% CI)	Rate (95% CI)	*p*-values^a^
Wheezing	26% (21–32%)	16% (11–21%)	**< 0.001**
Wheeze on exercise	20% (15–25%)	18% (13–23%)	0.200
Dry cough at night	23% (18–28%)	16% (11–21%)	**0.028**
Ever awakened due to wheezing	15% (11–19%)	7% (4–10%)	**0.001**
Current asthma (criteria-based)^b^	26% (21–31%)	16% (11–20%)	**< 0.001**
Severe asthma (criteria-based)^c^	13 (9–17%)	8% (4–11%)	0.064
Current use of asthma medication	26% (21–31%)	15% (10–19%)	**< 0.001**

Figures are the percentage of children with a positive response with the corresponding 95% confidence interval (95% CI)

^a^Mc Nemar’s test. Boldface denotes significant rate differences

^b^Defined by either (1) a doctor’s diagnosis of asthma and either respiratory symptoms or use of asthma medication in the previous 12 months, or (2) use of asthma medication and symptoms in the past 12 months even if no recall of prior doctor’s diagnosis

^c^Defined by four or more episodes of wheezing, or sleep disturbances (awakened more than once a week), or problems of speaking due to wheezing reported during the past 12 months (14)

Significantly more EP-born children with than without neonatal BPD had experienced wheezing and used asthma medication at 5–11 years of age, and there was still a marginal difference in medication rates at 11 years of age. A significantly lower fraction of the children born at GA > 28 weeks used inhaled corticosteroids (OR 0.41. 95% CI: 0.17, 0.99), otherwise respiratory symptoms or use of asthma medications at 5–11 years of age or at 11 years of age did not differ with GA (Table 5). Of the EP-born children, 18 (8%) had by definition severe asthma at 11 years of age. Severe asthma was associated with a parental history of asthma, in that seven of 38 (18%) EP-born children with a parental history of asthma had severe asthma compared to 11 of 191 (6%) EP-born children with no such history (*p* = 0.008). In multivariate regression analyses children of older mothers were less likely to report current respiratory symptoms while a parental history of asthma was associated with both current respiratory symptoms and a diagnosis of asthma (Table 4). Likewise, BPD with home oxygen therapy after discharge remained significantly associated with current asthma (Table 4). Adjusted for multiple hypotheses being tested, the variables closest to reaching the significance level were mother’s age for current respiratory symptoms (Bonferroni adjusted *p*-value 0.140) and parental asthma for both respiratory symptoms and current asthma (Bonferroni adjusted *p*-values 0.252 and 0.056, respectively).

When added to the multivariate model, lung disease diagnosed by a paediatrician at the five year follow-up was highly associated with current asthma at 11 years of age (OR 69.76, 95% CI 12.49, 389.54), but GA (OR 0.58, 95% CI: 0.36, 0.95) and BPD with home oxygen treatment (OR 13.18, 95% CI: 1.25, 138.84) also remained significant. Only 3/24 (13%) children with current asthma at 11 years of age were not considered to have lung disease at the five year follow-up.

## Discussion

At 5–11 years of age, the admission rate for the EP-born children was twice that of term-born controls, but occurred mainly for children with neurosensory disabilities and for surgical reasons, such as adeno-tonsillectomy or insertion of ear ventilatory tubes. Admissions for respiratory causes were rare, and neither BPD nor GA below 26 weeks at birth was associated with increased risk. Compared to the period 2–5 years of age, hospital admissions as well as respiratory morbidity had decreased, but admission rates, respiratory symptoms, current asthma, and use of asthma medication was still more common than in the control group. Statistical associations between most tested perinatal variables and the measures of morbidity during the study period were weak, evidenced by lack of significance after Bonferroni adjustments.

The strengths of this study were primarily the nationwide and population-based recruitment base and the longitudinal follow-up design that facilitated age-related assessments from early to mid-childhood in a country with free and unlimited access to health care for children. Although follow-up was not complete, important background information was available for all EP-born children, allowing proper assessment of representativeness. Thus, the number of stillbirths, postnatal deaths and perinatal differences between participants and non-participants could be completely accounted for. Participants tended to have less disabilities and a higher GA than those lost to follow-up, but a higher fraction had BPD. The number of eligible participants (*n* = 372) reflects the occurrence rate of EP deliveries, and was comparable to most similar studies [15, 16]. The 62% follow-up rate was disappointingly low when compared to previous follow-ups of this cohort, but reflects recent tendencies of increasing attrition rates in this type of research, in Norway [17], as well as internationally [16, 18]. Estimating GA was based on ultrasound at 17–18 weeks, performed within the frames of the established national free and all-encompassing program for antenatal care. Multiple perinatal variables were assessed for potential associations with the outcomes in regression models that utilised a dataset with limited numbers of participants. Thus, in order to prevent *type I* statistical errors, Bonferroni corrected *p*-values were reported. Regrettably, we were unable to recruit term-born control subjects for the complete cohort; however, individually matched term-controls were recruited based on the “next-born-subject” principle for a regional subsample representing 20% of the national population. Thus, the control group was considered unbiased and demographically representative for the complete cohort. Nevertheless, the small size of the control group reduced statistical power in the comparative analyses, and increased the risk of making *type II* errors, particularly as most outcome events were relatively rare.

Significantly more EP than term-born control children were admitted during the study period, which is in agreement with some [19, 20], but not all [21] previous studies. Admissions for respiratory diseases were quite uncommon, which is in agreement with a previous report [22], as was our finding that neither BPD [20] nor home oxygen treatment [23] were associated with admissions during 5-11 years of age. The data fit lung function findings that have previously been reported for this cohort when they were 11 years of age, in that neonatal BPD did not predict later airway obstruction [12]. Overall, these findings suggest that effects of extremely low GAs, BPD and duration of oxygen treatment have become less important for later pulmonary health as treatment of EP-born infants has improved.

At 11 years of age, 30% of the EP-born participants had ever been diagnosed with asthma, which was low compared to published rates of 37–46% among extremely low birth weight children at age 8–14 years [19, 24]. Regarding current wheezing, wheeze on exercise and current asthma, our findings were nearly identical to those of children of similar age born at GA below 26  weeks in the EPICure study [16], while current use of asthma medication was slightly less common (15% versus 25%). Our 16% rate of current asthma was lower than rates reported for somewhat younger [25, 26] and slightly older [21] extremely low birth weight children and for very low birth weight children of similar ages [27, 28]. However, comparing the prevalence of asthma between studies [16, 21, 25–28] is complicated due to lack of common diagnostic standards. Asthma is common also in the general paediatric population, and in a cohort of 10 year old children with BWs over 2000 grams born in Oslo in 1992 and 1993, 16.1% had ever been diagnosed with asthma, and 11.1% had current asthma [29]. In the present cohort, parental asthma was a strong predictor of asthma and wheezing when assessed at 11 years of age, but not at five years of age [10]. Thus, one may speculate that the occurrence of respiratory illness induced primarily by preterm birth decreases with age, while the relative importance of causes that are commonly implicated in unselected childhood populations (e.g. genetic determinants) increases with age.

For the children with neonatal BPD in this EP-born cohort, the rates for ‘asthma ever’ (35%) was similar to previous reports of 19–52% [18, 19, 30], while the rate of current asthma (20%) was in the lower end of reports ranging from 19% to 37% [16, 22, 31]. Current asthma at 11 years of age was not influenced by neonatal BPD, as was also observed by others [16, 18, 31, 32]. However, more children with BPD used asthma medication, both in this and other studies [18, 33], suggesting that they nevertheless might have more respiratory symptoms.

As regards development from the period 0–5 years of age to 5–11 years of age, the admission rates had declined significantly, but were still higher in the EP than term-born children, corresponding to Norwegian registry data comparing admissions between very preterm children and term-born children at similar ages [34]. Using The International Study of Asthma and Allergies in Childhood questionnaire, we found a significant reduction in respiratory symptoms from five to 11 years of age. This was encouraging, particularly as a large population based study of Western European children that utilised the same questionnaire reported a *higher* prevalence of current wheezing at age 13–14 years of age compared to 6–7 years of age (14.3% versus 9.6%) [14].

## Conclusions

In conclusion, respiratory morbidity reflected by hospital admissions and respiratory symptoms as reported by parents in validated questionnaires, were clearly less pronounced in mid-childhood than in early childhood in this nationwide cohort of EP-born children, but still more common than in a regionally recruited group of term-born children. There were few convincing associations between perinatal variables and measures of morbidity. Notably, children with low GA and a history of neonatal BPD did surprisingly well, and these variables did not influence admission rates or occurrence of current asthma in adjusted analyses.

## Abbreviations

BPD: Bronchopulmonary Dysplasia
BW: Birth Weight
EP: Extremely Preterm
GA: Gestational Age
ISAAC: International Study of Asthma and Allergies in Childhood
NICU: Neonatal Intensive Care Unit
OR (95% CI): Odds Ratio (95% Confidence Interval)
